# Acute hyperglycemia abolishes cardioprotection by remote ischemic perconditioning

**DOI:** 10.1186/s12933-015-0313-1

**Published:** 2015-11-18

**Authors:** Tamás Baranyai, Csilla Terézia Nagy, Gábor Koncsos, Zsófia Onódi, Melinda Károlyi-Szabó, András Makkos, Zoltán V. Varga, Péter Ferdinandy, Zoltán Giricz

**Affiliations:** Cardiometabolic Research Group, Department of Pharmacology and Pharmacotherapy, Semmelweis University, Nagyvárad tér 4, Budapest, 1089 Hungary; Pharmahungary Group, Szeged, Hungary

**Keywords:** Remote ischemic conditioning, Ischemia/reperfusion injury, Acute hyperglycemia, Autophagy, Nitrosative stress

## Abstract

**Background:**

Remote ischemic perconditioning (RIPerC) has a promising therapeutic insight to improve the prognosis of acute myocardial infarction. Chronic comorbidities such as diabetes are known to interfere with conditioning interventions by modulating cardioprotective signaling pathways, such as e.g., mTOR pathway and autophagy. However, the effect of acute hyperglycemia on RIPerC has not been studied so far. Therefore, here we investigated the effect of acute hyperglycemia on cardioprotection by RIPerC.

**Methods:**

Wistar rats were divided into normoglycemic (NG) and acute hyperglycemic (AHG) groups. Acute hyperglycemia was induced by glucose infusion to maintain a serum glucose concentration of 15–20 mM throughout the experimental protocol. NG rats received mannitol infusion of an equal osmolarity. Both groups were subdivided into an ischemic (Isch) and a RIPerC group. Each group underwent reversible occlusion of the left anterior descending coronary artery (LAD) for 40 min in the presence or absence of acute hyperglycemia. After the 10-min LAD occlusion, RIPerC was induced by 3 cycles of 5-min unilateral femoral artery and vein occlusion and 5-min reperfusion. After 120 min of reperfusion, infarct size was measured by triphenyltetrazolium chloride staining. To study underlying signaling mechanisms, hearts were harvested for immunoblotting after 35 min in both the NG and AHG groups.

**Results:**

Infarct size was significantly reduced by RIPerC in NG, but not in the AHG group (NG + Isch: 46.27 ± 5.31 % vs. NG + RIPerC: 24.65 ± 7.45 %, p < 0.05; AHG + Isch: 54.19 ± 4.07 % vs. 52.76 ± 3.80 %). Acute hyperglycemia *per se* did not influence infarct size, but significantly increased the incidence and duration of arrhythmias. Acute hyperglycemia activated mechanistic target of rapamycine (mTOR) pathway, as it significantly increased the phosphorylation of mTOR and S6 proteins and the phosphorylation of AKT. In spite of a decreased LC3II/LC3I ratio, other markers of autophagy, such as ATG7, ULK1 phopsphorylation, Beclin 1 and SQSTM1/p62, were not modulated by acute hyperglycemia. Furthermore, acute hyperglycemia significantly elevated nitrative stress in the heart (0.87 ± 0.01 vs. 0.50 ± 0.04 µg 3-nitrotyrosine/mg protein, p < 0.05).

**Conclusions:**

This is the first demonstration that acute hypreglycemia deteriorates cardioprotection by RIPerC. The mechanism of this phenomenon may involve an acute hyperglycemia-induced increase in nitrative stress and activation of the mTOR pathway.

## Background

Remote ischemic conditioning (RIC) is a clinically applicable cardioprotective intervention induced by intermittent occlusions and reperfusions on a remote organ e.g., a limb. It is proved to be highly effective against acute ischemia/reperfusion injury in animal models [1–4]. Due to its accessibility and simplicity, RIC was rapidly translated into different clinical situations of cardiovascular events [1, 3]. Despite the promising results of preceding animal studies, its infarct size reducing potential is equivocal in acute coronary syndrome patients [1]. Moreover, the largest randomized multi-center clinical trial including 1612 patients [Effect of remote ischemic preconditioning on clinical outcomes in patients undergoing coronary artery bypass graft surgery (ERICCA)] did not show any benefit on major adverse cardiac and cerebral events [5]. Reasons of these discrepancies are yet to be determined.

It has been shown that comorbidities (e.g., diabetes mellitus, hyperlipidemia) and comedications (e.g., angiotensin-converting enzyme inhibitors, statins) deteriorate cardioprotective effects of various conditioning stimuli (e.g., ischemic preconditioning and postconditioning) [2, 6, 7], however, only a few papers examined these chronic confounding factors in RIC so far. For example, Kiss et al. showed that remote ischemic perconditioning, i.e., RIC applied during a prolonged myocardial ischemia, (RIPerC) is not effective in a rat model of type 1 diabetes mellitus [8]. Similarly, the cardioprotective effect of RIC has been shown to be deteriorated in patients with type 1 and 2 diabetes mellitus [9]. Apart from being a major component of chronic metabolic diseases, hyperglycemia may also occur in acute situations (e.g., sympathetic overactivation during acute coronary syndrome) [10]. Hyperglycemia in nondiabetic patients (where hyperglycemia is unlikely to persist) is generally associated with adverse outcomes after an acute myocardial infarction [11]. It is not known whether this acute hyperglycemia is the cause of adverse outcomes or it only reflects the severity of the acute myocardial infarction [10]. Furthermore, it has been shown that hyperglycemia inhibits cardioprotection conferred by ischemic preconditioning or cardioprotection by various pharmacological agents [12–14]. However, it is not known whether acute hyperglycemia without pre-existing impaired glucose metabolism hinders cardioprotection by RIPerC.

Therefore, we aimed to investigate whether RIPerC-induced cardioprotection is affected by acute hyperglycemia and demonstrated for the first time in the literature that RIPerC failed to exert cardioprotection in acute hyperglycemia without pre-existing systemic metabolic disturbance. Furthermore, we have also shown that acute hyperglycemia increased nitrative stress and activated cardiac mechanistic target of rapamycine (mTOR) pathway, but not cardiac autophagy, which might be involved in the mechanism of the lost cardioprotection by RIPerC in acute hyperglycemia.

## Methods

This investigation conforms to the Guide for the Care and Use of Laboratory Animals published by the US National Institutes of Health (NIH publication No. 85–23, revised 1996), to the EU Directive (2010/63/EU) and was approved by the animal ethics committee of the Semmelweis University, Budapest, Hungary.

### In vivo experiments (Fig. 1)

220–280 g male Wistar rats were anaesthetized with 60 mg/kg pentobarbital. Since autophagy and cardioprotection are markedly influenced by fasting [15–17], animals were not fasted before enrolment. The absence of pedal reflex was considered as deep surgical anaesthesia. Electric activity of the heart was monitored (AD Instruments, Bella Vista, Australia). Blood pressure was measured in the carotid artery (AD Instruments, Bella Vista, Australia). Body temperature was maintained with a heat pad at physiological temperature (35.8–38.3 °C). Rats were ventilated with 10 mL/kg stroke volume at rate of 80 strokes/min (Ugo-Basile, Gemonio, Italy).

**Fig. 1:**
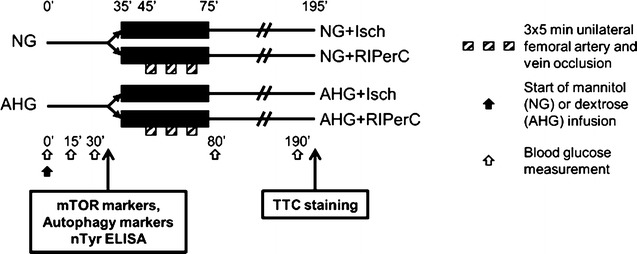
Experimental protocol. *NG* normoglycemia, *AHG* acute hyperglycemia, *Isch* ischemia only group, *RIPerC* remote ischemic perconditioning, *nTyr* 3–nitrotyrosine, *TTC* triphenyltetrazolium chloride

Rats were randomized into 2 groups: control normoglycemic (NG), and acute hyperglycemic (AHG). AHG animals received 50 % dextrose (vWR, Radnor, PA, US) infusion via tail vein from the start of the experimental protocol. A blood glucose level of 15–20 mM was reached within 5 min by an infusion rate of 150 µL/min. Then infusion rate was adjusted to maintain blood glucose levels between 15 and 20 mM throughout the entire protocol (0–60 µL/min, with an average of 50 µL/min), which was measured every 15 min (Accu-Check, Roche, Basel, Switzerland). In NG animals, an equal osmolarity, 46 % mannitol solution was administered (vWR, Radnor, PA, US, induction rate: 150 µL/min for 5 min, then 50 µL/min).

After 35 min of in vivo perfusion, half of the animals from normoglycemic and acute hyperglycemic groups were sacrificed, and hearts were excised, immersed readily in ice-cold Krebs-Henseleit solution until they were mounted. They were then perfused in Langendorff mode for 1 min with oxygenated (95 % oxygen/5 % CO_2_ gas mixture) Krebs-Henseleit solution (118 mM NaCl, 4.7 mM KCl, 1.2 mM MgSO_4_, 1.25 mM CaCl_2_, 1.2 mM KH_2_PO_4_, 25 mM NaHCO_3_ and 11 mM glucose) at 37 °C to wash out blood, as described earlier [18]. Then the hearts were snap-frozen in liquid nitrogen and stored at −80 °C until further experiments. The other half of the animals were further randomized into four groups: (1) control ischemic with normoglycemia (NG + Isch), (2) remote ischemic perconditioned with NG (NG + RIPerC), (3) ischemic with acute hyperglycemia (AHG + Isch), and (4) remote ischemic perconditioned with acute hyperglycemia (AHG + RIPerC). At 35′ of the study protocol, left anterior descending coronary artery (LAD) was occluded with a 6–0 polypropylene suture via median thoracotomy for 40 min. Occlusion was confirmed by ST segment elevation, arrhythmias and paling of the occluded area. RIPerC was induced by 3 cycles of 5 min occlusion and 5 min reperfusion of the right femoral vessels starting after 10 min of the LAD occlusion. Both the femoral artery and vein were occluded with a metal vessel clamp after isolation of the vessels from the surrounding connective tissue and femoral nerve. At the end of the 40 min index ischemia, reperfusion was induced by loosening the suture. At the end of the 120 min reperfusion, hearts were harvested for infarct size evaluation.

### Infarct size measurement

Hearts were excised after 120 min of reperfusion, perfused for 1 min with oxygenated Krebs-Henseleit solution in Langendorff mode. LAD was reoccluded, and the area at risk was negatively stained with Evans blue. For the assessment of viable myocardial tissue, 2 mm thick slices were cut and incubated in 1 % triphenyltetrazolium chloride (Sigma, St. Louis, MO, US) at 37 °C for 15 min. Slices were fixed in 4 % formalin for 16 h, weighed and scanned. Planimetric analyses were performed by two independent investigators with InfarctSize 2.4b software (Pharmahungary Group, Budapest, Hungary). Area at risk was expressed as the proportion of the left ventricular mass, and infarct size as the proportion of the area at risk mass.

### Arrhythmia analysis

An electrocardiogram was recorded throughout the entire experiment. Arrhythmia analysis was performed according to the Lambeth conventions, and arrhythmia incidence and duration scores were calculated [19].

### *Myocardial 3*-*nitrotyrosine measurement*

Free myocardial 3-nitrotyrosine was measured from left ventricular samples harvested at 35′ with 3-nitrotyrosine ELISA (Cayman, Ann Arbor, MI, US) according to the manufacturer’s protocol.

### Western blot

Left ventricular tissue was homogenized in radioimmunoprecipitation assay buffer (Cell Signaling, Danvers, MA, US) supplemented with protease inhibitor (Roche, Basel, Switzerland), sodium fluoride (Sigma, St. Louis, MO, US) and PMSF (Sigma, St. Louis, MO, US). Protein concentration of the homogenates was measured by Bicinchoninic Acid Assay kit (Thermo Fisher Scientific, Waltham, MA, US). Equal amount of protein (25 µg) was mixed with reducing 5× Laemmli buffer, loaded and separated in a 4–20 % precast Tris–glycine SDS polyacrilamide gel (Bio-Rad, Hercules, CA, US). Proteins were transferred onto a polyvinylidene difluoride membrane (Bio-Rad, Hercules, CA, US) at 350 mA for 2 h. Proper transfer was visualized with Ponceau staining (Sigma, St. Louis, MO, US). Membranes were blocked with 5 % BSA (Santa Cruz Biotechnology, Dallas, TX, US) in Tris-buffered saline containing 0.05 % Tween-20 (0.05 % TBS-T; Sigma, St. Louis, MO, US) at room temperature for 2 h. Membranes were probed with primary antibodies purchased from Cell Signaling (Danvers, MA, US) overnight at 4 °C (markers of mTOR pathway and its upstream modulators: phospho-mTOR [Ser2448]—#2971; mTOR—#2972; phospho-S6 [Ser235/236]—#2211; ribosomal S6—#2317; phospho-AKT [Ser473]—#9271; AKT—#9272; phospho-AMP-activated protein kinase α [AMPKα; Thr172]—#2535; AMPKα—#5831; phospho- extracellular signal-regulated kinase 1/2 [Erk1/2; Thr202/Tyr204]—#9106; Erk1/2—#9107; well-established markers of autophagy: microtubule-associated protein 1 light chain 3 A/B [LC3A/B]—#4108; beclin-1—#3495; SQSTM1/p62—#5114; phospho-UNC-51-like kinase 1 [p-ULK1; Ser555]—#5869; ULK1—#4773; autophagy-related gene 7 [ATG7]—#8558; Bcl-2/E1B-interacting protein 3 [BNIP3]—#3769; loading control: GAPDH—#5174), and with corresponding horseradish peroxidase-conjugated secondary antibodies (Cell Signaling, Danvers, MA, US) for 2 h at room temperature. Signals were detected with an enhanced chemiluminescence kit (Bio-Rad, Hercules, CA, US) by Chemidoc XRS+ (Bio-Rad, Hercules, CA, US). Antibodies detecting phosphorylated epitopes were removed with Pierce Stripping Buffer (Thermo Fisher Scientific, Waltham, MA, US) before incubation with antibodies detecting the total protein.

### *Triton X*-*100*-*insoluble SQSTM1/p62 Western blot*

Left ventricular tissue was homogenized with TissueLyser (Qiagen, Venlo, Netherlands) in a homogenation buffer containing 50 mM Tris, 150 mM NaCl, 1 mM EDTA, 10 % glycerol and 2 % Triton X-100 (pH 8.0) supplemented with protease inhibitor (Roche, Basel, Switzerland), sodium fluoride (Sigma, St. Louis, MO, US) and PMSF (Sigma, St. Louis, MO, US). Homogenates were centrifuged (10,000×*g*, 10 min, 4 °C) supernatant was carefully removed and discarded. Pellet was washed with the abovementioned homogenation buffer once again (10,000×g, 10 min, 4 °C). Then pellets were resuspended in sample buffer containing 62.5 mM Tris–HCl, 5 % glycerol and 1.3 % SDS. Protein concentration of the homogenates was measured by Bicinchoninic Acid Assay kit (Thermo Fisher Scientific, Waltham, MA, US). Equal amount of protein (20 µg) was loaded, separated and processed under reducing conditions as described above.

### Statistical analysis

Data was expressed as *mean* ± *standard error of mean*. *Student’s ttest* and *two*-*way ANOVA* with *LSD* as a post hoc *test* were used for statistical analyses in most cases, *Kaplan*–*Meier estimation* for evaluating mortality data, and *Kruskal*–*Wallis analysis* for analyzing scores. Statistical significance was accepted if p < 0.05.

## Results

### Acute hyperglycemia abolishes the protective effect of RIPerC

To investigate the effect of acute hyperglycemia on the efficacy of RIPerC, acute hyperglycemia was induced with a 50 % dextrose infusion during in vivo ischemia/reperfusion experiments. Blood glucose level was significantly elevated due to dextrose perfusion in both AHG + Isch and AHG + RIPerC groups compared to the corresponding NG group from at least the 15th min of perfusion [Table 1, preliminary results showed that acute hyperglycemia developed in 5 min (17.2 ± 1.8 mM, n = 4)]. Although baseline blood glucose appears to be higher than reported normal levels, it should be noted that baseline plasma glucose levels were measured in non-fasting animals which might have resulted in this relatively elevated, but still normoglycemic blood glucose level [20]. Elevated blood glucose levels did not influence the heart rate and blood pressure of the rats (Table 2). Until the end of the ischemic period, mortality rate was 11.1 % (1), 9.1 % (1), 30.0 %, (3) and 14.3 % (1), whereas total mortality was 0.0 % (0), 10.0 % (1), 14.3 %, (1) and 16.7 % (1) during reperfusion in Isch, RIPerC, AHG + Isch and AHG + RIPerC groups, respectively. Mortality was not significantly different between groups.

**Table 1:** Blood glucose (mM) is elevated in acute hyperglycemia

	0′	15′	30′		70′	190′
*NG*	8.6 ± 0.3	6.8 ± 0.2	8.4 ± 0.7	*NG* *+* *Isch*	9.2 ± 0.7	9.1 ± 1.1
				*NG* *+* *RIPerC*	9.2 ± 0.6	8.5 ± 0.5
*AHG*	8.5 ± 0.3	17.7 ± 1.0*	21.3 ± 2.1*	*AHG* *+* *Isch*	18.0 ± 1.3*	18.6 ± 0.6*
				*AHG* *+* *RIPerC*	19.9 ± 1.4^#^	18.4 ± 0.6^#^

*NG* normoglycemia, *AHG* acute hyperglycemia, *Isch* ischemia only group, *RIPerC* remote ischemic perconditioning

*p < 0.05 vs. corresponding time point of NG + Isch group

^#^p < 0.05 vs. corresponding time point of NG + RIPerC group. n = 5–10

**Table 2:** Acute hyperglycemia does not influence heart rate (HR) and mean arterial blood pressure (MABP)

	0′	15′	30′		40′	60′	80′	100′	120′	140′	160′	180′
HR (1/min)												
*NG*	428 ± 10	409 ± 12	415 ± 9	*NG* *+* *Isch*	438 ± 15	422 ± 12	430 ± 13	419 ± 10	411 ± 12	411 ± 18	425 ± 20	421 ± 14
				*NG* *+* *RIPerC*	419 ± 15	432 ± 14	418 ± 14	420 ± 8	428 ± 10	416 ± 9	427 ± 9	428 ± 13
*AHG*	420 ± 12	417 ± 8	403 ± 9	*AHG* *+* *Isch*	430 ± 15	425 ± 8	437 ± 11	422 ± 5	416 ± 5	415 ± 9	419 ± 8	410 ± 6
				*AHG* *+* *RIPerC*	410 ± 8	435 ± 10	409 ± 12	414 ± 16	408 ± 11	415 ± 11	406 ± 18	396 ± 18
MABP (mmHg)												
*NG*	111 ± 5	114 ± 7	114 ± 6	*NG* *+* *Isch*	116 ± 13	110 ± 10	109 ± 10	110 ± 9	109 ± 9	107 ± 8	108 ± 7	106 ± 11
				*NG* *+* *RIPerC*	111 ± 6	118 ± 6	109 ± 7	105 ± 7	107 ± 7	100 ± 8	105 ± 7	106 ± 7
*AHG*	112 ± 7	125 ± 8	119 ± 8	*AHG* *+* *Isch*	107 ± 8	103 ± 11	110 ± 7	105 ± 7	106 ± 7	101 ± 9	105 ± 6	108 ± 6
				*AHG* *+* *RIPerC*	98 ± 16	118 ± 13	106 ± 11	116 ± 10	109 ± 13	113 ± 11	106 ± 13	103 ± 12

*NG* normoglycemia, *AHG* acute hyperglycemia, *Isch* ischemia only group, *RIPerC* remote ischemic perconditioning

p > 0.05. n = 5–10

**Fig. 2:**
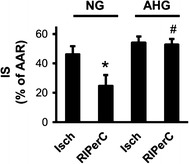
Acute hyperglycemia abolishes cardioprotective effect of RIPerC. Infarct size related to the AAR. *p < 0.05 vs. NG + Isch. ^#^p < 0.05 vs. NG + RIPerC. n = 5–10. *NG* normoglycemia, *AHG* acute hyperglycemia, *Isch* ischemia only group, *RIPerC* remote ischemic perconditioning, *IS* infarct size, *AAR* area at risk

Infarct size was significantly smaller in NG + RIPerC group compared to NG + Isch (24.65 ± 7.45 vs. 46.27 ± 5.31 %; p < 0.05; n = 7–10; Fig. 2), while RIPerC failed to decrease infarct size in AHG + RIPerC group when compared to AHG + Isch (52.76 ± 3.80 vs. 54.19 ± 4.07 %; p > 0.05; n = 5–6; Fig. 2). Furthermore, acute hyperglycemia *per se* did not aggravate cardiac necrosis (Fig. 2). There was no difference between the areas at risk of various groups (NG + Isch: 51.58 ± 1.65 %; NG + RIPerC: 45.13 ± 2.99; AHG + Isch: 49.44 ± 3.90; AHG + RIPerC: 43.64 ± 2.25 %; p > 0.05; n = 5–10).

### AHG exacerbates the incidence and duration of arrhythmias during ischemia

Arrhythmia analysis revealed that AHG compared to NG significantly increased the incidence and duration of arrhythmias during the whole myocardial ischemic period, but arrhythmia incidence and duration were not different during reperfusion (Table 3).

**Table 3:** Acute hyperglycemia exacerbates the incidence and duration of arrhythmias during ischemia

	0–35′		35–45′	45–75′	75–195′
Arrhythmia incidence scores					
*NG*	4.87 ± 1.28	*NG* *+* *Isch*	1.79 ± 0.73	2.47 ± 0.69	8.10 ± 3.01
		*NG* *+* *RIPerC*	3.09 ± 1.57	3.03 ± 0.98	6.56 ± 1.12
*AHG*	1.99 ± 0.45	*AHG* *+* *Isch*	14.06 ± 3.34*	23.17 ± 2.1*	8.79 ± 1.97
		*AHG* *+* *RIPerC*	10.69 ± 6.68	12.88 ± 6.87^#^	7.72 ± 3.19
Arrhythmia duration scores					
*NG*	3.65 ± 0.84	*NG* *+* *Isch*	1.59 ± 0.61	2.18 ± 0.57	7.58 ± 2.92
		*NG* *+* *RIPerC*	1.88 ± 0.73	2.18 ± 0.67	4.98 ± 0.82
*AHG*	1.99 ± 0.45	*AHG* *+* *Isch*	7.49 ± 1.58*	14.46 ± 2.84*	8.27 ± 1.95
		*AHG* *+* *RIPerC*	4.08 ± 1.43	8.98 ± 3.62^#^	7.71 ± 3.18

*NG* normoglycemia, *AHG* acute hyperglycemia, *Isch* ischemia only group, *RIPerC* remote ischemic perconditioning

*p < 0.05 vs. corresponding time point of NG + Isch group

^#^p < 0.05 vs. corresponding time point of NG + RIPerC group. n = 5–10

RIPerC did not alter arrhythmia scores from the time point it was applied, as compared to Isch group (Table 3). Furthermore, RIPerC did not decrease arrhythmia scores during acute hyperglycemia compared to AHG + RIPerC (Table 3).

### Acute hyperglycemia increases nitrative stress

**Fig. 3:**
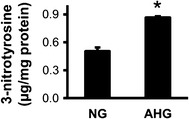
Acute hyperglycemia increases cardiac nitrative stress. 3-nitrotyrosine content of hearts of NG or AHG rats. *p < 0.05 vs. NG. n = 8. *NG* normoglycemia, *AHG* acute hyperglycemia

Increased oxidative and nitrative stresses are often implicated in the disruption of cardioprotective interventions. Therefore, 3-nitrotyrosine content, a marker of nitrative stress was measured in hearts of NG and AHG rats at 35′. Cardiac 3-nitrotyrosine was significantly elevated due to acute hyperglycemia (0.87 ± 0.01 vs. 0.50 ± 0.04 µg 3-nitrotyrosine/mg protein; p < 0.05; n = 8; Fig. 3).

### Acute hyperglycemia activates mTOR pathway

**Fig. 4:**
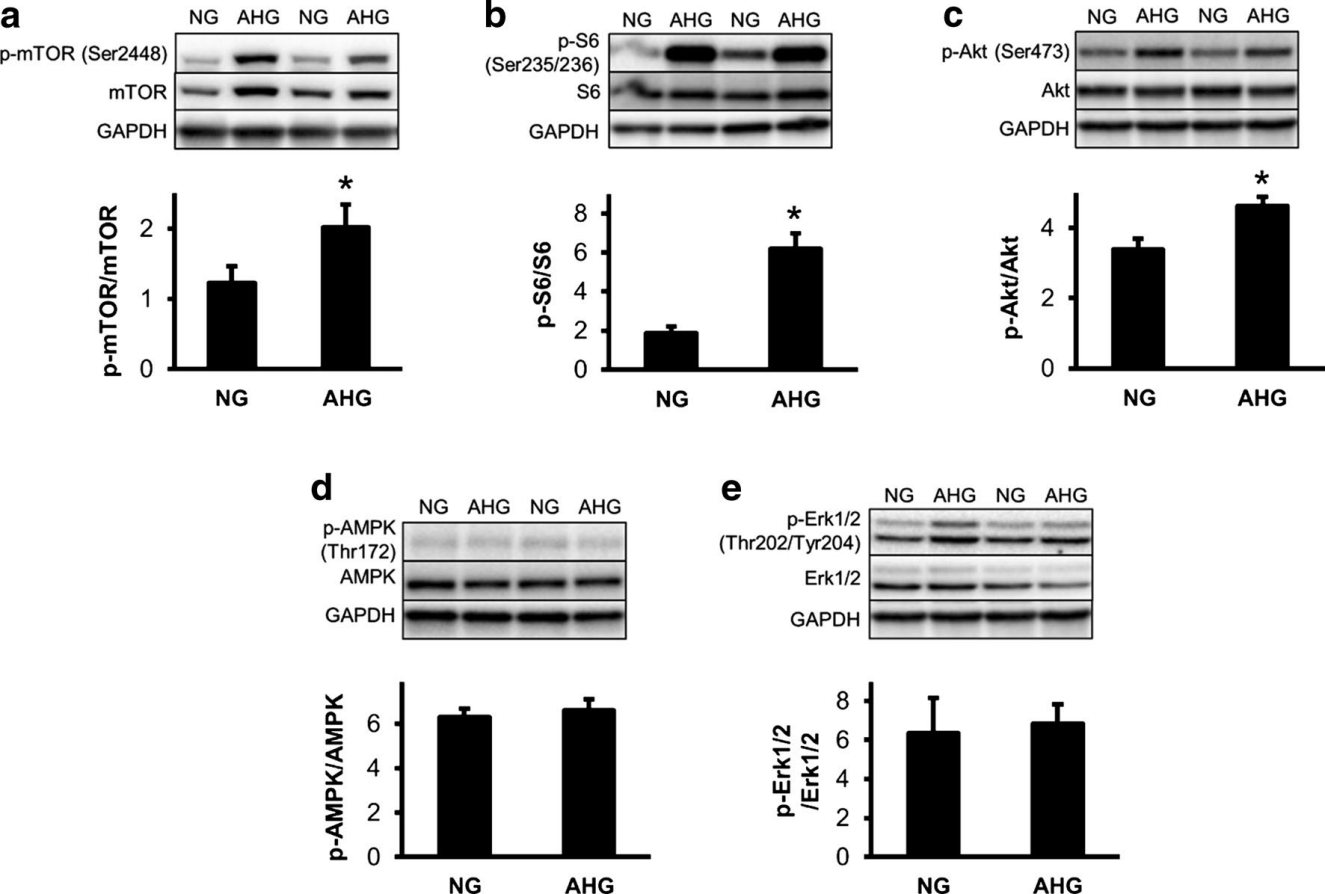
Acute hyperglycemia activates mTOR pathway. **a**–**e** Protein expression and/or phosphorylation of various mTOR-related proteins in the left ventricle. *p < 0.05 vs. NG. n = 7–9. *NG* normoglycemia, *AHG* acute hyperglycemia

Since the oxidative and nitrative stress have been previously shown to interact with mTOR pathway [21], we evaluated the expression and/or phosphorylation of mTOR pathway associated proteins. The phosphorylation of mTOR (Ser2448) and S6 (Ser235/236) was significantly elevated (Fig. 4a, b), which indicates that the activity of mTOR complex I was increased in AHG group. Phosphorylation of AKT at site Ser473 was also significantly elevated in AHG group (Fig. 4c), however, other mTOR regulators, such as phosphorylated AMPKα (Thr172) and Erk1/2 (Thr202/Tyr204) were unchanged in AHG group as compared to NG (Fig. 4d, e).

### Acute hyperglycemia does not influence autophagy

**Fig. 5:**
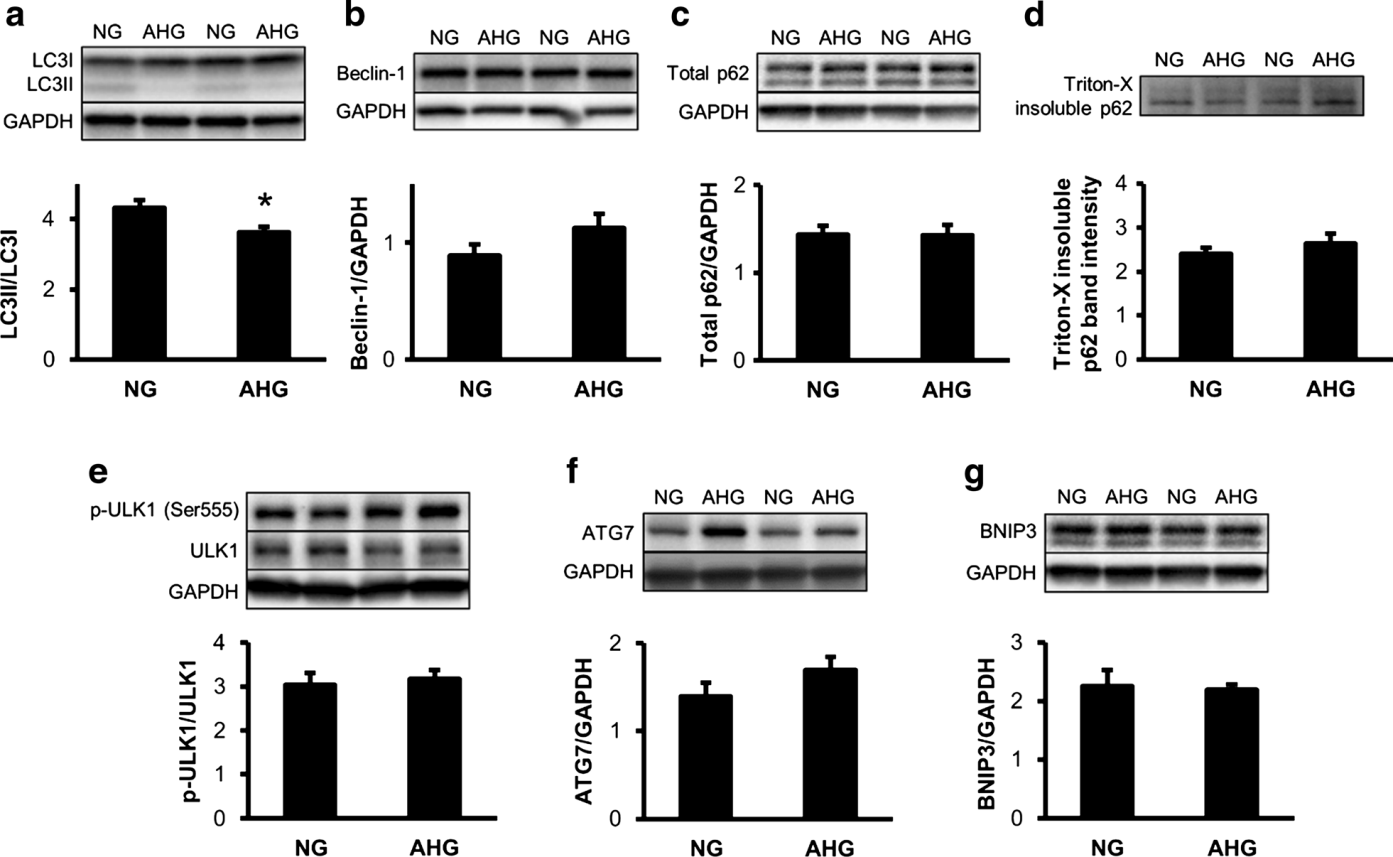
Acute hyperglycemia does not disturb autophagy. **a**–**g**. Protein expression and/or phosphorylation of various autophagy-related proteins in the left ventricle. *p < 0.05 vs. NG. n = 6–9. *NG* normoglycemia, *AHG* acute hyperglycemia

Since oxidative/nitrative stress and mTOR pathway have been shown to interact with autophagy, expression and/or phosphorylation of autophagy-related proteins were assessed in NG and AHG groups. LC3II/LC3I ratio was significantly decreased due to acute hyperglycemia (Fig. 5a), however, other autophagy-related proteins such as Beclin-1, total and Triton X-100-insoluble SQSTM1/p62, phospho-ULK1 (Ser555), ATG7 and BNIP3 were unchanged in AHG group (Fig. 5b–g).

## Discussion

We have demonstrated for the first time in the literature that acute hyperglycemia with no preceding diabetes mellitus abolished the infarct size limiting effect of RIPerC in an in vivo rat model with acute coronary occlusion and reperfusion. Furthermore, we have shown here that acute hyperglycemia did not influence autophagy, but increased nitrative stress in the heart plausibly through the activation of the AKT-mTOR pathway.

The major novelty of this study is that experimentally induced acute hyperglycemia with no preceding diabetes diminishes cardioprotective effect of RIPerC. This finding supports previous observations showing that other forms of cardioprotection may be affected by acute hyperglycemia. Kersten et al. described that cardioprotection by ischemic preconditioning is absent during acute hyperglycemia in dogs [13]. Similarly, acute hyperglycemia diminished cardioprotection conferred by isoflurane-induced preconditioning, however, it was reversible by increasing the minimum alveolar concentration in dogs [12]. It has been recently shown that hyperglycemia at admission does not deteriorate RIPerC, however, in this patient cohort, the presence of comorbidities, such as treated or untreated diabetes, have not been reported [22]. Although we showed here that acute hyperglycemia did not influence the extent of myocardial infarct size, a few studies have reported that acute hyperglycemia without any pre-exisisting pathophysiological conditions aggravate myocardial infarct size [13, 23, 24]. However, the majority of publications concludes that acute hyperglycemia *per se* do not change infarct size [25–27]. The seemingly contradictory findings may have been the result of applying different glucose concentrations, since reports suggesting harmful effects of acute hyperglycemia applied consistently higher glucose concentrations (i.e., over 30 mM).

The underlying mechanism of the loss of RIPerC-induced cardioprotection by acute hyperglycemia is not fully understood. Increased oxidative and nitrative stresses are implicated in the disruption of cardioprotective interventions by metabolic co-morbidities [2, 28–32], while conditioning stimuli such as RIC alleviates nitrative stress [28]. It was shown here that nitrative stress was also increased in acute hyperglycemia in rat heart in vivo, and similar results have been shown in isolated rat hearts perfused with hyperglycemic solution [33]. These findings clearly signal the pivotal role of excessive nitrative stress in the loss of cardioprotection in disturbed glucose homeostasis.

Oxidative and nitrative stresses have also been shown to directly disrupt autophagy (see for review: [34]). Therefore, we assessed cardiac autophagy and its regulatory pathways in acute hyperglycemia. However, we found that autophagy was unlikely to be disrupted, as only LC3II/LC3I ratio was significantly reduced, but other autophagy-related parameters were not. Although cardiac autophagy was not modulated, its most important regulator the mTOR pathway was largely activated by acute hyperglycemia. Since it has been shown that the inhibition of mTOR by rapamycine elicits cardioprotective effect in vivo [35, 36], and that RIC, while protecting the myocardium against ischemia, downregulates mTOR [37]. We hypothesize that the upregulated mTOR pathway might be responsible for this loss of cardioprotection by RIPerC in acute hyperglycemia. Furthermore, it was also been previously shown that under nutrient excess and oxidative stress, such as that seen in hyperglycemia, the mTOR pathway and its upstream modulator AKT are increasingly activated [38–40]. It is well established that activation of AKT upon reperfusion plays a central role in the mediation of cardioprotection conferred by ischemic pre-, post-, and remote conditioning (see for review: [41]). However, the cardioprotective effect of AKT activation before cardiac ischemia is controversial. Although genetic activation of AKT (24 h or 48 h prior to ischemia) protected the heart from ischemic insults [42, 43], more acute activation of AKT by SC79 and chronic AKT activation in *ob/ob* mice prior to ischemia did not confer protection against ischemia/reperfusion injury [44, 45]. We also demonstrated here that acute hyperglycemia-induced AKT activation prior to myocardial ischemia did not alter infarct size. These discrepancies could be explained by the fact that genetic activation of AKT induces an overwhelming alteration in cardiac gene expression profile [46] which might have not yet developed in our acute experiments. Moreover, we also showed that despite the acute hyperglycemia-induced activation of AKT, cardioprotective effects of remote ischemic perconditioning are lost. Similarly, it was previously reported that AKT activation prior to ischemia significantly interferes with protective stimuli, such as ischemic pre- and postconditioning [45, 47]. Therefore, one may conclude that the timing and the method of activation of AKT can profoundly influence its role in cardioprotection. Furthermore, AKT has a central role in the insulin signalling cascade and in the modulation of the mTOR pathway [48]. Here we evidenced an increased AKT in acute hyperglycemia, however, others found opposing trends in various cellular and in vivo models of hyperglycemia [26, 49, 50]. This discrepancy might be attributed to the substantial difference in the activation state of insulin signalling between model systems (i.e., missing insulin in STZ-treated animals or limited supply of insulin in cell cultures). Nevertheless, our current results demonstrate that AKT activation in an in vivo model with intact insulin and glucose homeostasis is detrimental on cardioprotection.

### Limitations

Here we evaluated the effect of acute hyperglycemia on the myocardium without ischemia or RIPerC. It is well established that ischemia and cardioprotective interventions significantly and dynamically influence autophagy and nitrative stress [28, 51, 52]. Thus, if such parameters are assessed after ischemia, i.e., in cardiac tissues with different level of exposure to ischemic insult, corresponding ischemic, border and remote zones, it would be unclear whether a possibly deteriorated autophagy and increased nitrative stress are causes or consequences of ischemia and/or reperfusion injury. Nevertheless, such experiments are still warranted to clarify the role of the mTOR pathway, autophagy and nitrative stress in the loss of RIPerC in acute hyperglycemia.

## Conclusions

In conclusion, here we have shown evidence for the first time in the literature that the cardioprotective effect of RIPerC is lost in acute hyperglycemia. The mechanism of this phenomenon may involve an acute hyperglycemia-induced increase of nitrative stress and activation of the AKT-mTOR pathway, but not the disruption of cardiac autophagy. This data suggests that the efficacy of RIPerC might be compromised in clinical settings with acute hyperglycemia.
